# Associations between mother-preschooler attachment and maternal depression symptoms: A systematic review and meta-analysis

**DOI:** 10.1371/journal.pone.0204374

**Published:** 2018-10-02

**Authors:** Shaylea Badovinac, Jodi Martin, Camille Guérin-Marion, Monica O’Neill, Rebecca Pillai Riddell, Jean-François Bureau, Rebecca Spiegel

**Affiliations:** 1 Department of Psychology, York University, Toronto, Ontario, Canada; 2 School of Psychology, University of Ottawa, Ottawa, Ontario, Canada; 3 Department of Psychiatry Research, The Hospital for Sick Children, Toronto, Ontario, Canada; 4 Department of Psychiatry, University of Toronto, Toronto, Ontario, Canada; National Center for Child Health and Development, JAPAN

## Abstract

The current study aimed to systematically review and meta-analyze concurrent and longitudinal associations between maternal depression symptoms and mother-child attachment during the preschool period (aged 2 to 7 years) as assessed using the coding systems by Cassidy and Marvin (1992) and Main and Cassidy (1988). The review was pre-registered with PROSPERO (International Prospective Register of Systematic Reviews; Registration number CRD42017073417) and was conducted in accordance with PRISMA (Preferred Reporting Items for Systematic Reviews and Meta-Analyses) guidelines. A total of 7,969 records were screened and 18 articles were deemed as eligible for inclusion in the review. Studies were reviewed using qualitative synthesis techniques and, where appropriate, meta-analysis. Qualitative synthesis indicated that mothers of disorganized/controlling children most consistently reported the highest levels of depressive symptoms, both concurrently and longitudinally. The association between disorganized/controlling child attachment and concurrent maternal depressive symptoms was significant (*n* = 1,787; *g* = 0.27, 95% CI [0.13,0.40]), and was not moderated by sample type, child gender, or risk of bias. Findings of a relationship between child attachment insecurity and maternal depressive symptoms must be qualified due to significant within-study heterogeneity and publication bias. Results suggest that maternal depressive symptoms may confer risk for disorganized/controlling attachment during the preschool period.

## Introduction

Attachment theory posits that infants are biologically predisposed to forming close bonds with their primary caregivers as a strategy to ensure that their fundamental needs are met [1]. This bond, known as the attachment relationship, represents a unique aspect of the caregiver-child relationship that goes beyond the infant’s basic needs and the caregiver’s ability to provide for those needs. Specifically, the attachment bond is shaped by the dyadic patterns of caregiver and child behaviour in a distressing context. The formation of a secure attachment relationship is supported by the presence of a parent or caregiver who consistently recognizes and responds sensitively to the child’s distress signals [2,3]. In the short-term, a secure caregiver-infant attachment relationship provides a context in which a distressed infant can seek out their caregiver with the expectation of being comforted and supported [2]. In the long-term, this relationship supports the child in learning the skills needed to independently manage their own social and emotional functioning [4,5].

Various methodologies have been developed for measuring and describing individual differences in attachment behaviours during infancy and beyond. In general, attachment researchers who study infants’ regulatory style with a primary caregiver [1] have used the Strange Situation Procedure (SSP; [2,6]) as the gold standard assessment measure of infant attachment. Subsequent to pioneering work in infant attachment initiated by Bowlby and Ainsworth [1,2], more recent work has moved into the study of attachment during the preschool years (i.e., 2–7 years). These years represent a key period of transition with regards to child development, as the child’s social world begins to extend beyond family, and the child forms friendships with peers and other adults at daycare or school. Rapid advances in cognitive abilities, language, and emotional knowledge equip the child as they take on these new challenges in their social environment, by supporting their understanding, communication, and regulation of emotion [7]. Accordingly, the preschool period is also a time of transition in terms of attachment behaviours [8]. Preschool-aged children are not as readily distressed by minor stressors (e.g., brief separation from caregiver) as compared to infants, but they continue to rely on their attachment figures to a greater extent compared to older children and adolescents. Thus, attachment dynamics during this important developmental period are expected to differ from earlier and later periods, although their function may be the same.

To inform this newer area of research, two attachment coding systems were developed by Main and Cassidy [9] and shortly thereafter by Cassidy and Marvin (with the MacArthur Attachment Working Group) [10]. Both systems are considered to be analogues of the SSP [11](i.e., moderately distressing paradigms) with developmentally appropriate modifications, such as longer caregiver-child separations, and were developed for children aged two and a half to four and a half [10] and five to seven [9] respectively. Similarly to the infant SSP, these systems yield attachment classifications of secure (B), insecure-avoidant (A), or insecure-ambivalent (C). However, both systems differ from the SSP in their operationalization of disorganized attachment. The Cassidy and Marvin system [10] for children aged two and a half to four and a half years describes a controlling/disorganized spectrum which is further differentiated into controlling-punitive, controlling-caregiving, controlling-mixed, and behaviourally-disorganized profiles [12]. Conversely, the Main and Cassidy system [9] for children aged five to seven years includes classifications of controlling (D; includes controlling-punitive and controlling-caregiving) and unclassifiable (i.e., behaviours that do not fit into other indices, including behaviourally-disorganized).

With the creation of measures for effective assessment of attachment during the preschool years that parallel the gold standard measure of infancy, a sizeable body of research has accumulated in the past four decades assessing attachment among preschool-aged children and the predictors, correlates, and antecedents of attachment during this period. Yet, to our knowledge, to date no syntheses have organized the results of preschool attachment studies via a systematic review or meta-analysis. In light of this gap in our understanding, the current study aimed to focus on a more integrated understanding of the relationship between preschool attachment and maternal mental health, an established and critical correlate of infant attachment [13].

### Maternal mental health and child attachment

Maternal mental health problems have been named as a major public health challenge by the World Health Organization owing to the high global prevalence of mental health challenges among women during the pre- and post-natal periods [14]. Depressive disorders are the most commonly diagnosed psychological disorders for mothers [14] and are among the most common classes of mental illness within the general global population [15].

Maternal mental health challenges have been hypothesized to affect mother-child attachment by undermining a mother’s ability to engage in sensitive caregiving, a key predictor of early attachment behaviours [3]. A caregiver who has difficulty regulating negative emotions- a symptom of depression and other mood disorders [16]- may face attentional barriers that limit their ability to identify, process and respond appropriately to the behaviours and emotions of their children [17], tasks which are core features of sensitive caregiving [2]. Supporting this, research investigating the impact of depression on parenting broadly has found depressed mothers to exhibit more negative, hostile, and disengaged affect and behaviours and fewer positive behaviours during interactions with their children [18], interactions styles that are in direct opposition to sensitive caregiving.

Much of the research investigating direct associations between maternal mental health and infant and child attachment has focused on the impact of parental depressive symptoms or clinical depression. This research was the focus of a meta-analysis by Atkinson and colleagues [13], who found a significant relationship between maternal depressive symptomology and attachment security (*r* = .18), and found that this relationship was significantly stronger among clinically depressed samples, compared to community samples. This review was based on evidence from 15 studies of primarily mother-infant dyads. Only three of these studies focused on the preschool period (> 24 months of age), with one study using one of the preschool attachment classification systems, and these three studies represented three distinct clinical populations. In light of this and given the fact that new data from larger-scale longitudinal studies (e.g., National Institute of Child Health and Human Development Study of Early Child Care and Youth Development; NICHD SECCYD) have since accrued, an updated synthesis is needed to gain a more complete understanding of how maternal depressive symptoms relate to attachment specifically during the preschool period. Moreover, there is reason to expect that the impact of maternal mental health challenges during the preschool period may be different from infancy due to changes in the amount of time many children spend in the sole care of the primary caregiver as the child transitions to school or daycare.

### The current study

The objective of the current study was to systematically review and meta-analyze concurrent and longitudinal associations between maternal depression symptoms and attachment in preschool-aged children (2 to 7 years) as assessed using the coding systems by Cassidy and Marvin [10] and Main and Cassidy [9]. We also aimed to investigate how these associations varied as a function of sample type (normative vs. clinical), child age, and how attachment outcomes were operationalized (e.g., A/B/C/D vs. secure/insecure vs. organized/ disorganized and controlling). Based on previous research in infants, we expected to identify significant associations between maternal depressive symptoms and attachment outcomes, particularly with regards to attachment insecurity. We also expected associations between maternal depressive symptoms and child attachment outcomes to be stronger among clinical samples, compared to normative samples [13].

## Method

### Search strategy

This systematic review and meta-analysis was conducted in accordance with the Preferred Reporting Items for Systematic Reviews and Meta-Analyses (PRISMA)[19] guidelines and the review protocol was registered with PROSPERO prior to data extraction (Registration number: CRD42017073417) [20]. Our methodology differed slightly from the published protocol due to there being insufficient papers examining maternal anxiety or stress and preschool attachment that met our inclusion criteria, thus the review focused only on depression and depressive symptomology. Please see PRISMA check-list provided in S1.

The search strategy was developed with the assistance of an academic librarian at the Hospital for Sick Children in Toronto, Ontario, Canada. Search terms were selected and paired by identifying key terms related to the construct of caregiver-child attachment, children between the ages of two and seven, and the Main and Cassidy [9] and Cassidy and Marvin [10] attachment classification systems. The systematic search was conducted in CINAHL, Embase, Medline, and PsycINFO and was last updated on June 14^th^ 2018 (see S2 for an example of our search strategy).

### Inclusion and exclusion criteria

To be eligible for inclusion, studies were required to: a) include a measure of maternal depression (symptoms or diagnosis) administered to caregivers and b) report on direct relationships between caregiver depression symptoms and attachment as rated by the Main and Cassidy [9] or Cassidy and Marvin [10] coding systems (or report sufficient data for post-hoc calculations). Studies investigating depression specifically in the parenting role (e.g., using the Parenting Stress Index; [21]) were not included in order to minimize heterogeneity, and studies that evaluated the efficacy of maternal mental health interventions were only retained if they included and reported on a control (i.e., non-intervention) group or reported baseline (i.e., pre-intervention) data.

Studies published prior to 1985 were excluded, as the earliest documented reference to the preschool attachment coding systems was dated 1985 (cited in [22]). In addition, studies meeting any of the following exclusion criteria were also discarded: language other than English or French, non-human attachment, non-attachment, attachment examined in children less than or equal to 2 years of age or older than 7 years of age, review articles (or commentaries, abstracts, case studies, dissertations), examined attachment using a different preschool attachment assessment procedure (e.g., Attachment Story Stem Battery [23]) or a different coding system (e.g., Preschool Assessment of Attachment [24]).

### Study selection

The systematic search identified 14,568 records. Following the removal of duplicates, the titles and abstracts of 7,969 records were screened by four independent reviewers and irrelevant studies were excluded according to an *a priori* search algorithm. Thirty percent of abstracts were double-coded and overall agreement on double-coded abstracts was 84%. All disagreements were resolved through consensus. Inclusion criteria had to be evident from the abstract, due to the large number of abstracts eligible for review. However, if an abstract was unclear (e.g., age, attachment measure/coding system) and: 1) was authored by individuals identified to contribute to the development of the preschool attachment coding manuals [9,10]; or 2) was authored by key authors in the field of child attachment; or; 3) featured National Institute of Child Health and Development (NICHD) data, it was retained for full-text review (further detail provided in S3).

**Fig 1:**
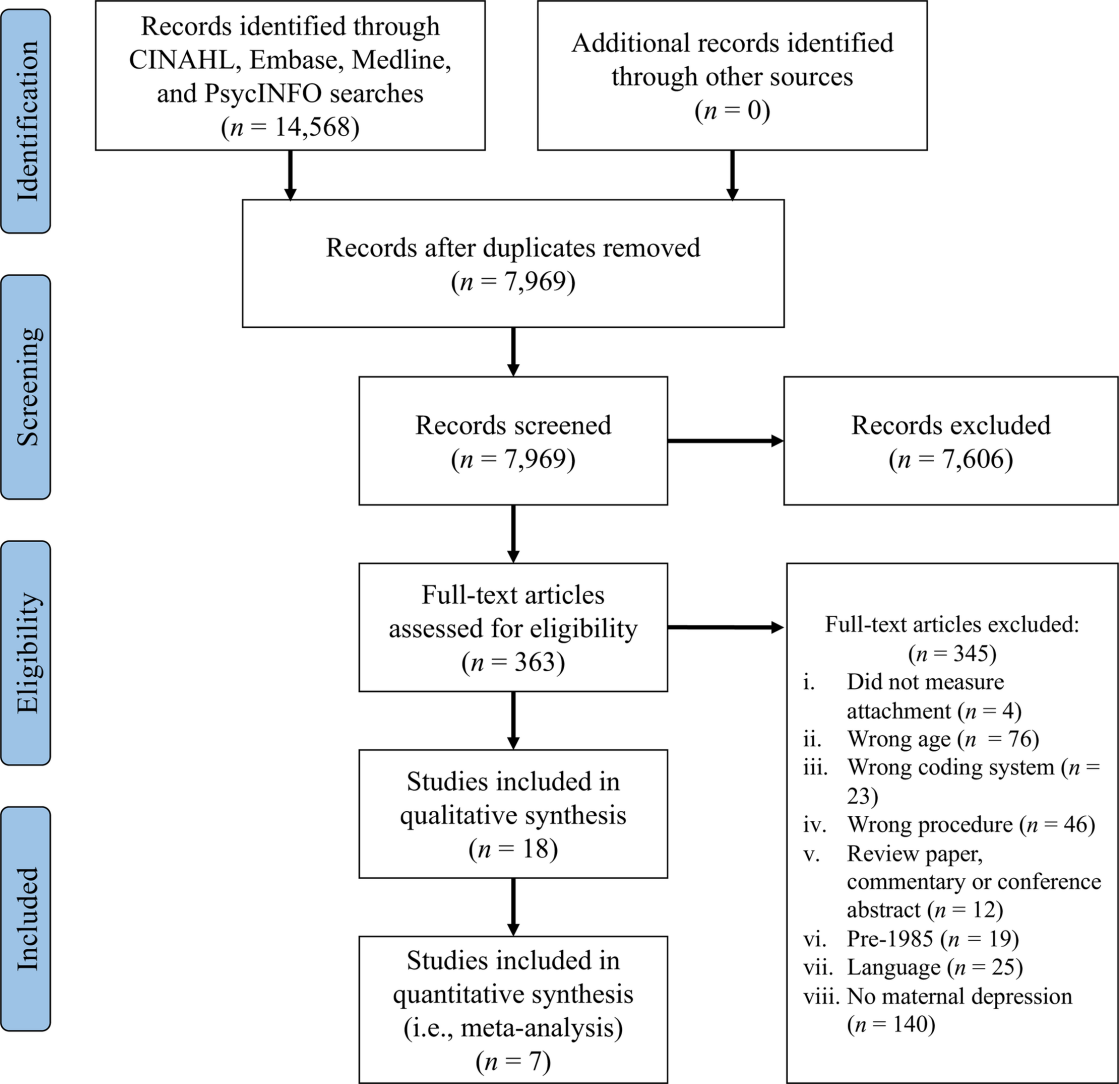
Included study flow chart following PRISMA guidelines.

In total, 363 articles met criteria for full-text review based on the aforementioned procedure. Eighteen articles were identified as meeting inclusion criteria for the current study and thus were included in the narrative qualitative synthesis, and seven of these articles provided sufficient information to be included in the meta-analysis (Fig 1).

### Data extraction

Reviewers used a standardized extraction form to collect the following data from each included study: demographic characteristics (community/ clinical sample, gender distribution of sample, sample socioeconomic status), sample size, country, methodology for assessment of maternal depression (i.e., measure used, time between child attachment and maternal mental health assessment), operationalization of attachment outcomes used in the current analyses (e.g., A/B/C/D categorizations, secure vs. insecure dichotomy, organized vs. disorganized/controlling dichotomy), and data related to associations between child attachment and maternal depression (including covariates, where applicable). Effect sizes were extracted, if available. In addition, any group-level quantitative data that could be used to calculate additional effect sizes (e.g., means and standard deviations, proportions, etc.) were extracted. For example, in cases where a study analyzed group differences among secure and insecure groups but also reported descriptive data on maternal depression for each attachment category (A/B/C/D), these descriptive data were extracted and used to calculate effect sizes for other attachment categorizations (e.g., organized vs. disorganized/controlling comparisons, A/B/C/D comparisons). In the event that no quantitative data were available, study authors were contacted by email and additional information was requested, unless another included study reported quantitative data from the same sample. Studies from which quantitative data could not be obtained were qualitatively synthesized.

### Risk of bias

The present study used an adapted version of the NIH National Heart, Lung, and Blood Institute’s Quality Assessment Tool for Observational Cohort and Cross-Sectional Studies [25] as there is currently no gold standard measure for assessing risk of bias in observational studies [26]. This tool, which evaluates sources of bias in study design, was adapted to include four additional items from the Downs and Black [27] and Crombie [28] checklists which evaluate the quality of reporting for each article. The adapted risk of bias tool is included as S4, with six key items prioritized for an additional risk of bias judgment (see below). Thirty percent of articles were double-coded for risk of bias, and inter-rater reliability was strong (intra-class correlation = 0.95). Discrepancies were resolved through discussion.

For each study, reviewers indicated whether each source of bias was present, absent, or not applicable (e.g., retention rate for a cross-sectional study). A risk of bias score for each study was calculated as the proportion (%) of sources of bias present out of the total number of sources of bias applicable to the study. Thus, studies with higher scores had a higher risk of bias in reporting or study design. Risk of bias scores were used as a covariate in meta-analyses and reported on in order to quantify the quality of evidence presented by each study.

In line with NIH recommendations [25], an additional and more holistic risk of bias judgment was formed for each article based on consensus between reviewers. These consensus-driven judgments were based on review of six items from the aforementioned risk of bias tool, which were prioritized due to their relative importance in assessing studies’ methodological integrity. These six items pertained to: study power, validity and reliability of measures, blinding of attachment coders, longitudinal participant retention, and consideration of key confounding variables (i.e., child gender, family socioeconomic status). For each study, raters discussed all six items to determine the extent to which the study managed each potential source of bias. Based on this discussion, an overall risk of bias judgment was assigned to each article (“Higher” or “Lower” risk of bias).

### Data analysis

Data analysis involved a three-step process. First, preliminary calculations were conducted, which involved calculating effect sizes using group-level data reported within each study. Second, quantitative synthesis was used to statistically combine effect sizes across studies, in cases where sufficient data was available to do so (conditions to be described below). Third, qualitative synthesis was used to summarize results only in cases where the available data was not sufficient for quantitative synthesis. Each of these steps will be described in detail below.

#### Calculation of effect sizes

The first step in data analysis involved using the reported data within each study to compute standardized effect sizes. Group means and standard deviations and Pearson correlations were used to calculate the standardized mean differences between groups using Cohen’s *d*. In cases where proportions were reported (e.g., proportion of maternal depression diagnoses across attachment categories), log odds ratios were calculated and converted to Cohen’s *d*. In cases where data for each attachment category were reported separately (e.g., A/B/C/D), pooled means and standard deviations or combined proportions were calculated in order to determine effect sizes based on a secure vs. insecure or organized vs. disorganized/controlling dichotomy. All calculations were conducted based on formulae found in work by Lipsey and Wilson [29].

The resulting data were then organized based on three grouping variables. First, results were categorised based on child age at assessment (2–5 years vs. 5–7 years). Second, results within these overarching categories were organized based on whether they reported on concurrent (i.e., within three months) or longitudinal (i.e., > 3 months) associations between maternal depression symptoms and child attachment. Third, results were further sub-stratified according to the attachment operationalization used. These included: four-way classification (Avoidant, Secure, Ambivalent, Disorganized/Controlling [ABCD]), Secure vs. Insecure dichotomy (Secure vs. the combined Avoidant, Ambivalent, and Disorganized/controlling categories [SI]), Organized vs. Disorganized/controlling dichotomy (Disorganized/controlling vs. the combined Avoidant, Secure, Ambivalent categories [OD]), and Secure vs. Insecure-organized vs. Disorganized/controlling trichotomy [SID]. Thus, the organization of the results from the studies (i.e. outcome category) reflected three factors: Child Age at Assessment (2–5 years vs. 5–7 years), Temporal Analysis (Longitudinal vs. Concurrent), and Attachment Operationalization (ABCD vs. SI vs. OD vs. SID). Following the organization of results based on these three factors, outcomes were synthesized either qualitatively or quantitatively.

Our goal was to meta-analyze results within each of the outcome categories described above and investigate the impact of moderating variables using meta-regression. However, owing to a low number of studies and high degree of sample redundancy within certain outcome categories, our results represent a combination of quantitative (i.e., meta-analysis, meta-regression) and qualitative syntheses. A description of our decisions as well as the analytic techniques used in each case will now follow.

#### Quantitative synthesis

Effect sizes within each outcome category were quantitatively synthesized when two conditions were met. First, only effect sizes for secure vs. insecure or organized vs. disorganized/controlling attachment categorizations were quantitatively synthesized. This decision was made in order to limit the number of quantitative analyses performed on each sample, and in accordance with previous research that has identified insecure attachment generally and disorganized attachment specifically as being associated with worse developmental outcomes [30,31]. Second, and with the goal of minimizing Type I error in light of sample redundancy, effect sizes for secure vs. insecure or organized vs. disorganized/controlling attachment categorizations were only quantitatively synthesized when at least three effect sizes were available to combine.

Quantitative synthesis involved meta-analysis and meta-regression. Hedges’ *g* was used as the outcome effect size in meta-analyses. Therefore, effect sizes for results included in meta-analyses were transformed from Cohen’s *d* to Hedges’ *g* [32]. In the context of the current study, both Cohen’s *d* and Hedges’ *g* represent the standardized mean difference between attachment groups with respect to maternal depression, however Hedges’ *g* corrects for positive bias [33]. Interpretation of both Hedges’ *g* and Cohen’s *d* follow the same convention, with ≤ 0.2, 0.5, and ≥ 0.8, representing small, medium, and large effects, respectively [34,35]. Random-effects models were used for the meta-analyses since between-studies differences beyond sampling error (e.g., differences due to methodological differences) were anticipated. Meta-analyses were conducted using the *metafor* package [36] in *R* [37] and the meta-analysis dataset is available in S5.

Cochran’s *Q* and *I*^2^ were used to assess heterogeneity among effect sizes included in the meta-analyses. While Cochran’s *Q* is used to detect the presence of heterogeneity, *I*^*2*^ quantifies the extent of heterogeneity. Cochran’s *Q* represents the weighted sum of squared differences between each individual study’s effect and the pooled effect across studies [38]. A significant Cochran’s *Q* value suggests the presence of statistically significant between-study variation [39]. *I*^2^ was introduced as a supplement to Cochran’s *Q* and represents the percentage of the total variation across studies that is due to heterogeneity [38]. *I*^2^ values of 25%, 50%, and 75% have been purported to correspond with low, moderate, and high degrees of heterogeneity [38].

Egger’s test [40] was used to assess funnel plot asymmetry, which may be a sign of publication bias (i.e., the bias that makes studies with positive findings more likely to be published). Egger’s test involves a regression of the standard normal deviate (individual effect size divided by standard error) onto the estimate’s precision (inverse of standard error) [40]. A significant result indicates that the regression intercept is significantly different from zero and suggests that publication bias may be present. However, previous research has indicated that under certain circumstances, funnel plot asymmetry may be indicative of other sources of heterogeneity other than publication bias [40]. Accordingly, only when Egger’s test revealed significant plot asymmetry, contour-enhanced funnel plots were inspected [41], which are funnel plots with shaded areas of statistical significance. In these plots, a white area in the middle of the funnel plot represents non-statistically significant effects, and shaded areas towards the edges and outside the funnel represent statistically significant effects. An over-representation of studies in shaded areas (i.e., areas of statistical significance) is suggestive of publication bias.

Redundancy across the selected studies occurred as a result of studies investigating different waves of the same longitudinal study (e.g., NICHD, Moss, MAVAN) or studies investigating different iterations of a larger sample (e.g., only including dyads with complete data on selected variables). Within each meta-analysis, each unique sample was only represented by one effect size. In cases where multiple effect sizes were available from the same sample, effect sizes from studies with methodologies that most closely resembled the methodologies of the other included studies in the meta-analysis were selected in order to minimize heterogeneity among studies. For example, when effect sizes from multiple waves of the same study were available (e.g., 3-year-old or 5-year-old waves of Moss sample), we selected effect sizes from the wave in which the child’s age (at attachment assessment) was most similar to the other included studies for a given meta-analysis. Furthermore, when effect sizes from different iterations of the same sample (e.g., sub-analyses of NICHD sample) were available, the effect size from the study with the largest sample size was used.

The influence of moderator variables on meta-analyses was investigated using meta-regression. Moderator variables included risk of bias, sample type (i.e., normative, clinical), mother or family’s socioeconomic status (SES) and child gender, and were selected in line with previous meta-analyses of psychopathology and child attachment [3,13,31]. Risk of bias was operationalized as the study’s risk of bias score (proportion ranging from 0–100; described previously). Sample type was designated as normative or clinical. Clinical samples included studies in which the mother and/or child had received a clinical diagnosis, or which over-sampled dyads with clinical risk factors (e.g., prenatal cocaine/opiate exposure). Mother or family socioeconomic status was operationalized as High/Middle or Low. In line with previous work, studies that did not report on socioeconomic status were labeled as High/Middle [31]. Child gender was operationalized as the percentage of boys in the sample.

#### Qualitative synthesis

Qualitative synthesis was conducted in instances where the quantitative data provided was insufficient for quantitative synthesis (and no data was provided in response to our email requests). Qualitative synthesis involved a description of the general direction and magnitude of findings from each relevant study in turn, followed by an integration of findings in the form of a brief summary. To avoid redundancy due to overlapping samples, the summary integrated findings across attachment categorizations within a given age group and time frame (i.e., concurrent/longitudinal).

## Results

Eighteen studies were included in the present review, and seven of these studies were included in the meta-analyses.

### Study characteristics

Table 1 provides an overview of key study characteristics. Below is a summary.

**Table 1:** Study characteristics.

Reference	Research Group / Sample	Country	*N*^a^	Sample Type	Maternal Mental Health Outcome(s)	Child Age at Attachment Assessment	Maternal Mental Health Outcome Measure(s)	Attachment categorizations used in current syntheses	Risk of Bias Score^b^	Risk of Bias Judgement^c^
Campbell et al., 2004 [51]	NICHD SECCYD	USA	1077	Normative	Depression	2–5 years	CES-D	ABCD SI	26.67	Lower
Cyr & Moss, 2001 [56]	Moss	Canada	91	Normative	Depression	5–7 years	BDI	ABCD	50.00	Higher
Dubois-Comtois & Moss, 2004 [59]	Moss	Canada	38	Normative	Depression	5–7 years	BDI	SID	35.71	Higher
Graffi et al., 2018 [43]	MAVAN	Canada	304	Clinical/Risk	Depression	2–5 years	CES-D	OD	13.33	Lower
Manassis, Bradley, Goldberg, Hood, & Swinson, 1994 [44]	Unique	Canada	20	Clinical/Risk	Both	2–5 years	BDI	ABCD SI	71.43	Higher
Milan, Snow, & Belay, 2009 [49]	NICHD SECCYD	USA	938	Normative	Depression	2–5 years	CES-D	SI	42.86	Lower
Mills-Koonce, Gariepy, Sutton, & Cox, 2008 [50]	NICHD SECCYD	USA	1140	Normative	Depression	2–5 years	CES-D	ABCD	46.67	Lower
Moss, Bureau, Cyr, Mongeau, & St-Laurent, 2004 [48]	Moss	Canada	151	Normative	Depression	2–5 years	BDI	ABCD SI OD	42.86	Lower
Moss, Cyr, & Dubois-Comtois, 2004 [57]	Moss	Canada	242	Normative	Depression	5–7 years	BDI	ABCD	20.00	Lower
Moss, Rousseau, Parent, St-Laurent, & Saintonge, 1998 [58]	Moss	Canada	121	Normative	Depression	5–7 years	BDI	ABCD	46.67	Higher
O’Connor, Bureau, McCartney, & Lyons-Ruth, 2011 [12]	NICHD SECCYD	USA	1140	Normative	Depression	2–5 years	CES-D	ABCD SI OD	21.43	Lower
Seifer et al., 2004 [42]	MLS	USA	742	Clinical/Risk^b^	Depression	2–5 years	BDI	ABCD SI OD	40.00	Lower
Spieker & Crittenden, 2010 [55]	NICHD SECCYD	USA	306	Normative	Depression	2–5 years	CES-D	ABCD	14.29	Higher
Stevenson-Hinde & Shouldice, 1995 [45]	Unique	United Kingdom	78	Normative	Both	2–5 years	IDA	ABCD SI OD	64.29	Higher
Stevenson-Hinde, Shouldice, & Chicot, 2011 [46]	Stevenson-Hinde	United Kingdom	98	Normative	Both	2–5 years	HADS	ABCD SI	46.15	Higher
Toth, Rogosch, Manly, & Cicchetti, 2006 [52]	Cicchetti	USA	117	Clinical/Risk	Depression	2–5 years	DIS-III-R	ABCD SI OD	20.00	Lower
Toth, Rogosch, Sturge-Apple, & Cicchetti, 2009 [53]	Cicchetti	USA	99	Clinical/Risk	Depression	2–5 years	DIS-III-R	ABCD	46.67	Lower
Wazana et al., 2015 [47]	MAVAN	Canada	301	Clinical/Risk	Depression	2–5 years	CES-D	OD	20.00	Lower

Note: NICHD SECCYD, National Institute for Child Development Study of Early Child Care and Youth Development; MLS, Maternal Lifestyle Study; MAVAN, Maternal Adversity, Vulnerability and Neurodevelopment; CES-D, Center for Epidemiological Studies Depression Scale; BDI, Beck Depression Inventory; IDA, Irritability, Depression, and Anxiety Scale; HADS, Hospital Anxiety and Depression Scale; DIS-III-R, Diagnostic Interview Schedule; ABCD, Avoidant/Secure/Ambivalent/Disorganized and Controlling; SI, Secure/Insecure; OD, Disorganized and controlling/Organized; SID, Secure/Insecure-Organized/Disorganized and controlling.

^a^ Sample size used in focal analyses relevant to the present review.

^b^ Primarily low socioeconomic-status sample.

^c^ Detail regarding determination of Risk of Bias Scores and Risk of Bias Judgments is provided in methodology section.

#### Sample description

The majority of studies were conducted in the United States (*k* = 8) and Canada (*k* = 8), with the remaining studies being conducted in the United Kingdom (*k* = 2). Collectively, more than half of the studies were based on the NICHD SECCYD or Ellen Moss’ French-Canadian longitudinal sample (*k* = 5, respectively). Collectively, the studies represent eight unique samples, including four normative samples and four clinical/risk samples. Only one study was based on a primarily low-SES sample. The remaining studies featured middle- to high-SES samples. The majority of studies (*k* = 14) assessed attachment in two- to five-year-olds and four assessed attachment in five- to seven-year-olds.

All but one study (*k* = 17) examined mother-child attachment exclusively. Of these, seven studies examined biological mother-child dyads, and the remaining ten did not specify whether participants were limited to biological mother-child dyads. One study examined caregiver-child attachment (which could have included fathers, maternal relatives, non-relatives, foster parents and/or non-primary caregivers), but was still primarily composed (97%) of biological mothers [42].

#### Attachment categorizations

Operationalizations of attachment outcome varied between studies, and most studies reported results based on multiple different operationalizations. The following numbers reflect the attachment operationalizations from each study that were used for the current study (in qualitative or quantitative synthesis): Fourteen studies used the four-way classification system (ABCD; secure, avoidant, ambivalent, disorganized/controlling), nine studies used secure vs. insecure comparisons (B vs. A/C/D/Controlling; SI), seven used organized vs. disorganized/controlling comparisons (A/B/C vs. D/Controlling; OD) comparisons, and one compared secure (B), insecure-organized (A/C) and disorganized/controlling (D/Controlling) groups (SID).

#### Risk of bias

**Fig 2:**
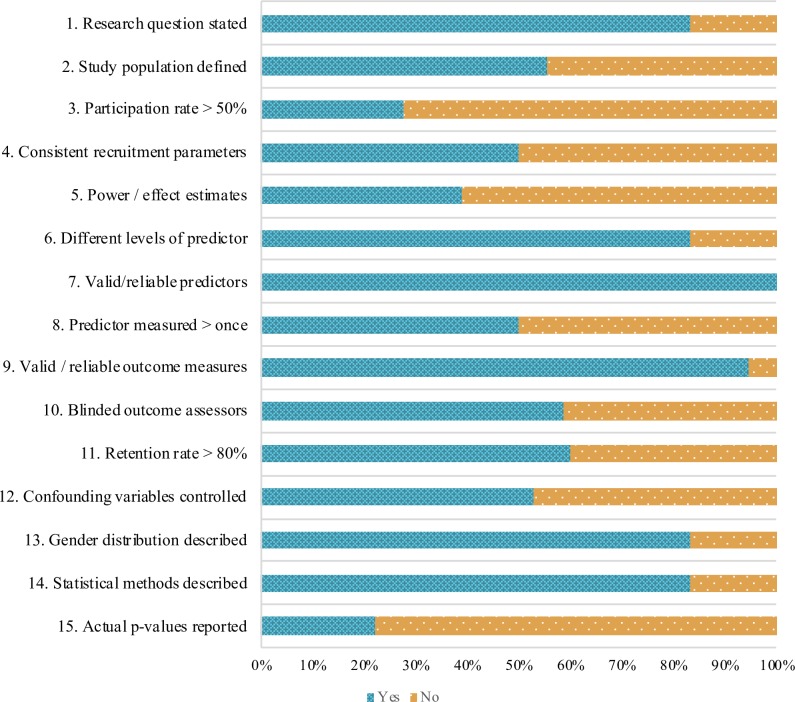
Risk of bias scores. Bar graph illustrating proportion of studies that fulfilled each risk of bias consideration.

The average risk of bias score was 37.17%. Scores ranged from 13.33% [43] to 71.43% [44]. Item-level analysis revealed that the criteria that were most rarely met were: reporting exact p-values (met by 22% of studies), reporting a participation rate of at least 50% (met by 28% of studies) and reporting a power analysis or effect size estimates (met by 39% of studies). Risk of bias scores were negatively correlated with publication year (*r* = -.72, *p* = 0.001), suggesting that more recent studies tended to have a lower risk of bias. When the risk of bias of each study was considered holistically based on key items (risk of bias judgment), seven studies (39%) were judged to have a “Higher” risk of bias and 11 studies (61%) were judged to have a “Lower” risk of bias. Fig 2 illustrates the proportion of studies that received credit for each risk of bias item.

The syntheses from the 18 studies are presented below. As aforementioned, results are organized based on three factors: Child Age at Assessment (2- to 5-year-olds vs. 5- to 7-year-olds), Temporal Analysis (Longitudinal vs. Concurrent), and Attachment Operationalization (ABCD vs. SI vs. DO vs. SID). Within each outcome category, results were summarized either qualitatively or quantitatively (meta-analysis and meta-regression), depending on the data available. Table 2 provides a succinct overview of all the results described below.

**Table 2:** Summary of syntheses.

	Articles analyzed	Synthesis technique	Summary of results
**1. Maternal depressive symptoms and attachment in 2- to 5-year-olds**			
**1.1. Maternal depressive symptoms and attachment in 2- to 5-year-olds: Concurrent associations**			
1.1.1. Secure vs. Insecure	[12, 44, 45, 46, 48, 49, 52]	Quantitative	Small effect indicating higher depression levels among mothers of insecure children (*g* = 0.3, *p* = .01, 95% CI [0.06, 0.55]), but result may be influenced by publication bias. Higher effect sizes among clinical samples and samples with more boys.
1.1.2. Organized vs. Disorganized/controlling	[12, 45, 47, 48, 52]	Quantitative	Small effect indicating higher depression levels among mothers of disorganized/controlling children (*g* = 0.27, *p* = .0001, 95% CI [0.13, 0.40]).
1.1.3. A/B/C/D Categorization	[12, 45, 48, 50, 52, 53]	Qualitative	In three of four samples, mothers of disorganized/controlling children had significantly higher depression levels than mothers of secure children, with effect sizes in small range. Suggests consistent differences in maternal depression levels between secure and disorganized/controlling categories.
**1.2. Maternal depressive symptoms and attachment in 2- to 5-year-olds: Longitudinal associations**			
1.2.1. Secure vs. Insecure	[42, 51]	Qualitative	One study found no relationship (based on depression symptoms at child age four months), one study found a relationship between intermittent and chronic symptom elevations and insecurity. Suggestive of relationship when persistent symptoms present.
1.2.2. Organized vs. Disorganized/controlling	[42, 43, 47	Qualitative	No significant relationships found.
1.2.3. A/B/C/D Categorization	[42, 50, 51, 55]	Qualitative	In one sample, no differences were found (based on depression symptoms at child age four months). In another sample, mothers in disorganized/controlling dyads reported the highest depression levels across the first three years of life (small effect size) and were more likely to have a history of persistent elevated depression scores. Suggests differences between secure and disorganized/controlling groups which may vary as a function of time between assessments or sample type.
**2. Maternal depressive symptoms and attachment in 5- to 7-year-olds**			
**2.1. Maternal depressive symptoms and attachment in 5- to 7-year-olds: Concurrent associations**			
2.1.1. A/B/C/D Categorization	[57, 58]	Qualitative	No relationships found. Trends indicated higher depression levels reported by mothers of behaviourally-disorganized children (small effect).
2.1.2. Secure vs. Insecure-Organized vs. Disorganized/Controlling	[59]	Qualitative	No relationships found.
**2.2. Maternal depressive symptoms and attachment in 5- to 7-year-olds: Longitudinal associations**			
2.2.1. A/B/C/D Categorization	[56, 57]	Qualitative	No relationships found. Trends indicated more symptoms reported by mothers of behaviourally-disorganized children and least reported by mothers of secure children.

### Maternal depressive symptoms and attachment in 2- to 5-year-olds

Thirteen studies assessed attachment in children aged 2 to 5 years, using the Cassidy and Marvin [10] system [12,42–53].

#### 1.1. Maternal depressive symptoms and attachment in 2- to 5-year-olds: Concurrent associations

1.1.1. Secure vs. Insecure: Quantitative synthesis. Seven studies reported on differences between secure and insecure two- to five-year-old children with regards to concurrent maternal depression symptoms. Since two of the studies were based on the NICHD SECCYD [12,49], the study with the larger sample size [12] was retained for the meta-analysis.

The overall weighted mean effect size of differences between two- to five-year-olds with secure vs. insecure attachment in terms of concurrent maternal depressive symptoms was calculated based on a total sample of 1,604 mother-child dyads, drawn from two clinical samples [44,52] and four community samples [12,45,46,48]. Three of the studies were judged to have a lower risk of bias [12,48,52] and three were judged to have a higher risk of bias [44–46].

**Fig 3:**
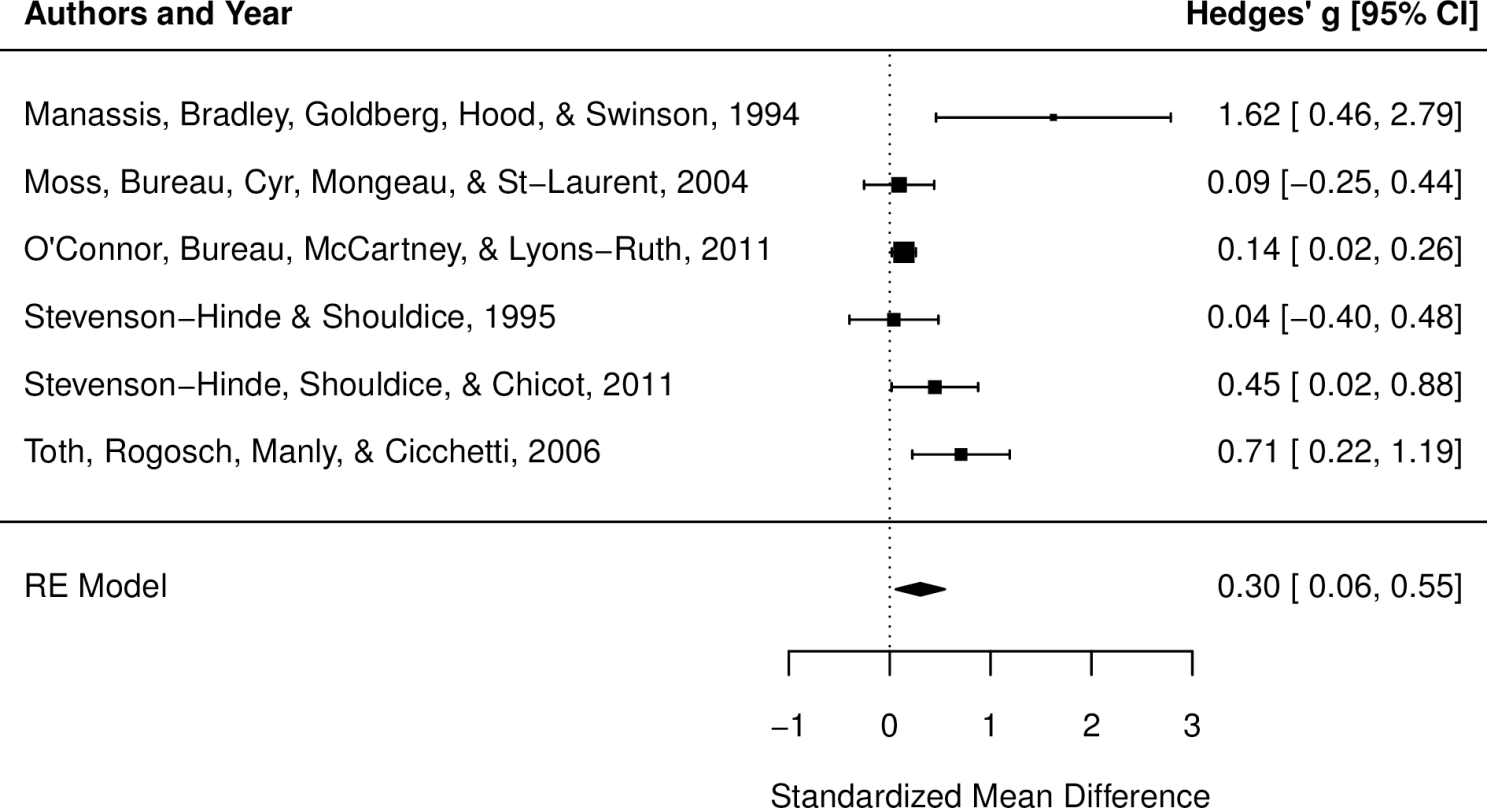
Forest plot for meta-analysis of maternal depressive symptoms and concurrent attachment insecurity in 2- to 5-year-olds. Hedge’s g point estimates are depicted by filled squares, with square sizes reflecting the relative weight of each study’s effect size in the analysis. The filled diamond reflects the summary effect size. RE = Random effects model. If a square or error bars cross 0, this indicates no difference between mothers of secure and insecure preschoolers. Squares to the right of zero indicate higher depression levels among mothers of insecure preschoolers, relative to mothers of secure preschoolers.

**Fig 4:**
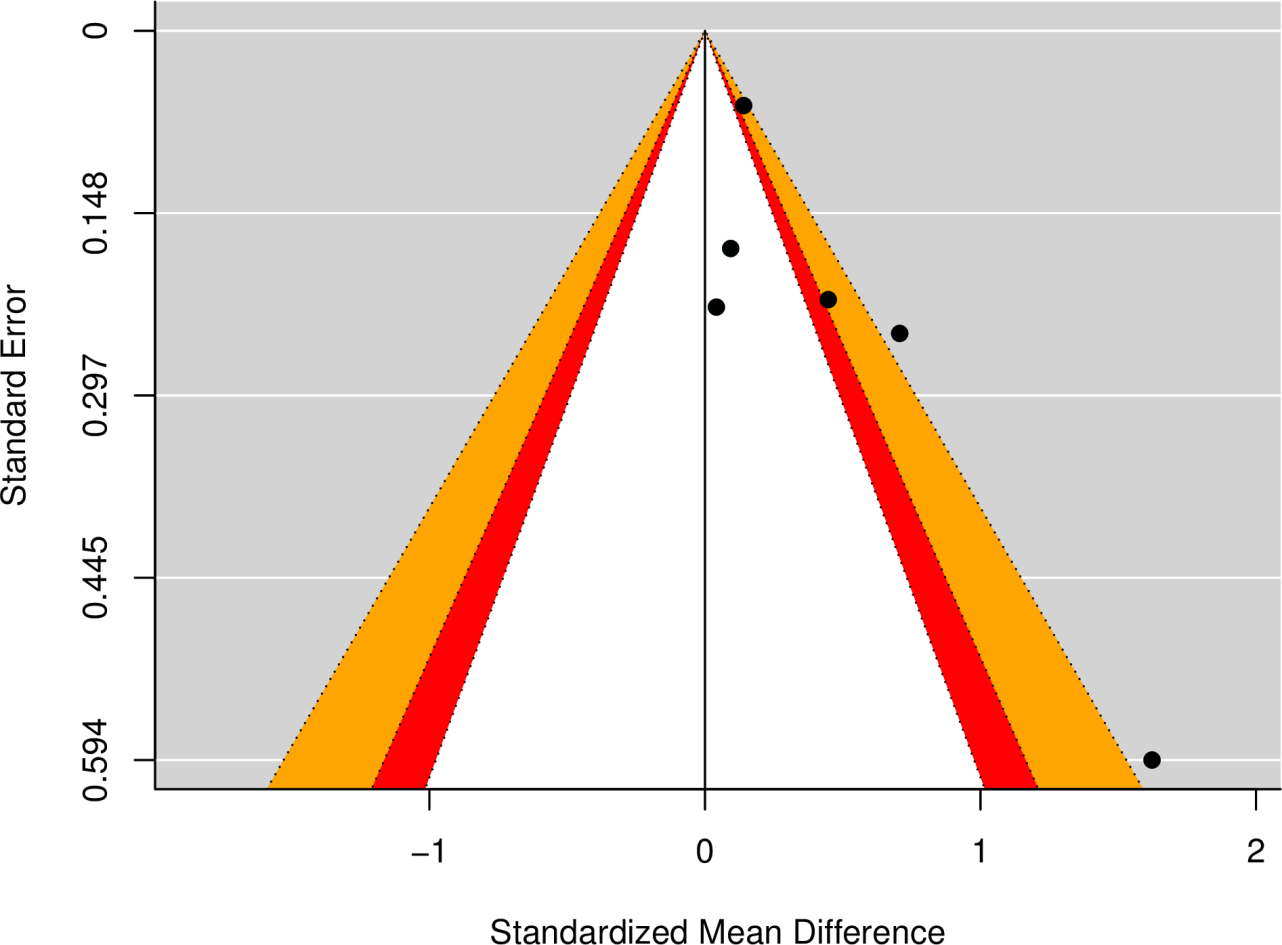
Contour-enhanced funnel plot for meta-analysis of maternal depressive symptoms and concurrent attachment insecurity in 2- to 5-year-olds. Each dot represents an included study in the meta-analysis. An over-representation of dots in the shaded (non-white) areas of statistical significance is suggestive of publication bias.

Results of the meta-analysis indicated a small effect, *g* = 0.30, *p* = .01, 95% CI [0.06, 0.55] (see Table 3), suggesting that depression levels were higher among mothers of insecure children, in comparison to mothers of secure children (Fig 3). There was a moderate degree of heterogeneity among study effects (*Q* = 13.00, *p* = .02, *I*^2^ = 59.40%; see Table 3). Results of Egger’s test [40] indicated significant funnel plot asymmetry (*z* = 2.27, *p* = .02), and accordingly, a contour-enhanced funnel plot was inspected to screen for evidence of publication bias (Fig 4). This revealed an over-representation of studies in the shaded significance areas, which is suggestive of publication bias [41].

**Table 3:** Summary statistics from meta-analyses of maternal depressive symptoms and concurrent attachment in 2- to 5-year-olds.

Comparison	*g*	SE	z	95% CI	*p*	Q	I^2^ (%)	*df*
Insecure vs. Secure (*n* = 1,604)	0.30	0.12	2.44	[0.06, 0.55]	.01	13.00*	59.40	5
Disorganized/controlling vs. Organized (*n* = 1,787)	0.27	0.07	3.87	[0.13, 0.40]	.0001	3.34	6.19	4

Note: *g* = Hedges’ *g; Q* = Cochran’s heterogeneity statistic; Q; I^2^ = percentage of total variation across studies that is due to heterogeneity.

*p < .05

The moderator analyses revealed significant results for sample type (Q_b_ = 8.76, *p* = 0.003) and child gender (Q_b_ = 4.50, *p* = 0.03), indicating that between-groups differences in maternal depression symptoms were larger in clinical samples (*g* = 0.84, *k* = 2) compared to normative samples (*g* = 0.15, *k* = 4), and in samples with a larger percentage of boys (*g* = 0.06). The moderator analysis for risk of bias scores was not significant (Q_b_ = 0.08, *p* = 0.78). A moderator analysis could not be conducted for SES, as all included studies in this analysis were based on middle-to-high SES samples.

1.1.2. Organized vs. Disorganized/controlling: Quantitative synthesis. The overall weighted mean effect size of differences between two- to five-year-old children with organized vs. disorganized/controlling attachment with regards to concurrent maternal depressive symptoms was calculated based on 1,787 mother-child dyads. Data was drawn from five studies representing five independent samples [12,45,47,48,52]. Two of these were clinical samples [47,52], while the other three were community samples [12,45,48]. One study was judged to have a higher risk of bias [45] and the remaining four were judged to have a lower risk of bias [12,47,48,52].

**Fig 5:**
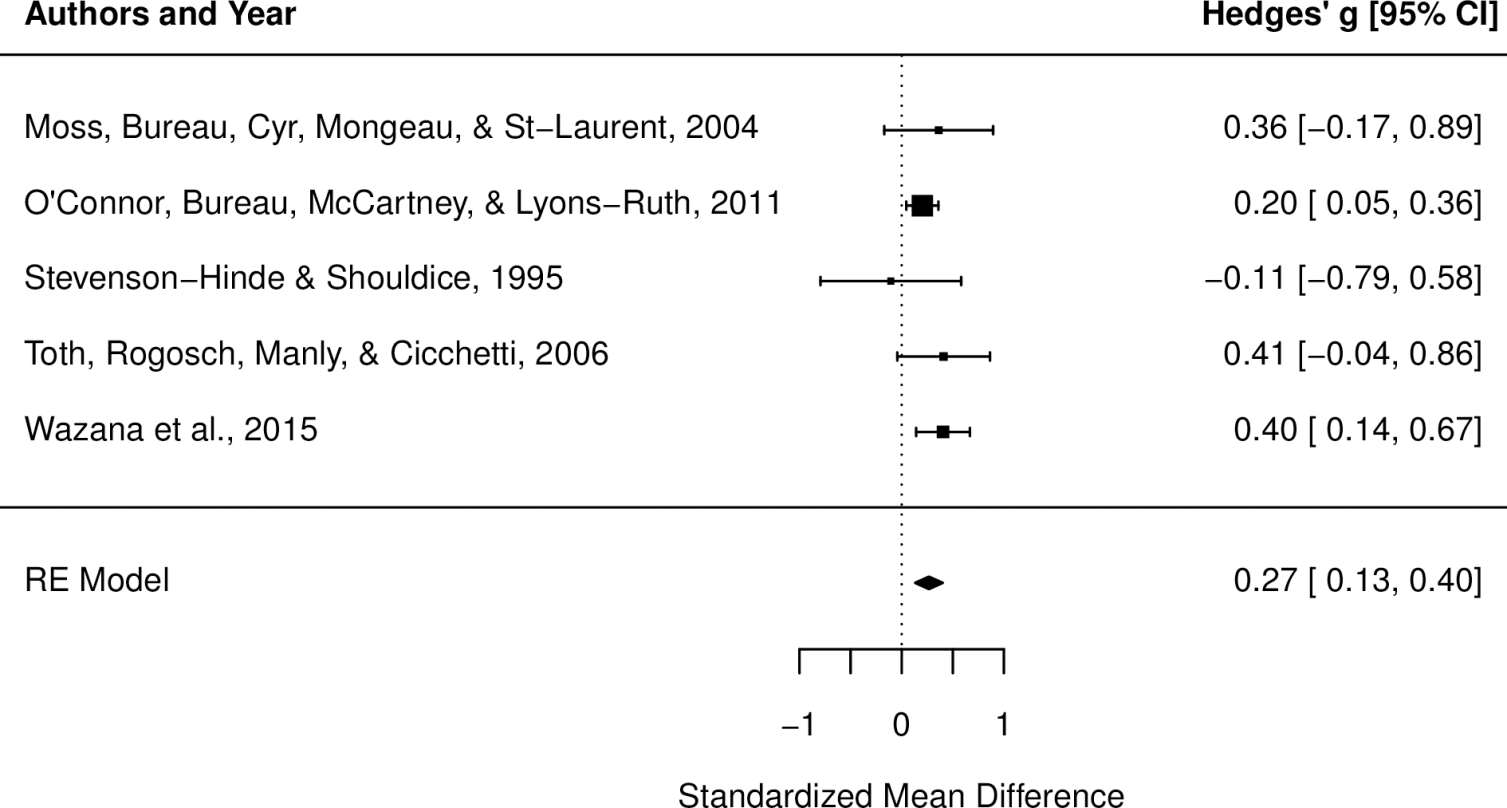
Forest plot for meta-analysis of maternal depressive symptoms and concurrent attachment disorganization in 2- to 5-year-olds. Hedge’s g point estimates are depicted by filled squares, with square sizes reflecting the relative weight of each study’s effect size in the analysis. The filled diamond reflects the summary effect size. RE = Random effects model. If a square or error bars cross 0, this indicates no difference between mothers of organized and disorganized preschoolers. Squares to the right of zero indicate higher depression levels among mothers of disorganized preschoolers, relative to mothers of secure preschoolers.

Results of the meta-analysis indicated a small effect, *g* = 0.27, *p* = .0001, 95% CI [0.13, 0.40] (see Table 3). This indicates that depression levels were higher among mothers of disorganized/controlling children, in comparison to organized children (Fig 5). Egger’s test [40] revealed no significant funnel plot asymmetry (*p* = 0.75), suggesting that the overall effect is robust. There was a low degree of heterogeneity among study effects, indicating that the evidence supporting this result was relatively consistent (*Q* = 3.34, *p* = .50, *I*^2^ = 6.19%).

Meta-regression results suggested that between-groups differences were not moderated by risk of bias scores (Q_b_ = 0.70, *p* = 0.40), sample type (Q_b_ = 3.27, *p* = 0.07), or the proportion of boys in the sample (Q_b_ = 2.18, *p* = 0.14). A moderator analysis could not be conducted for SES, as all included studies in this analysis were based on middle-to-high SES samples.

1.1.3. A/B/C/D Categories: Qualitative synthesis. Six studies reported on concurrent associations between maternal depressive symptoms and attachment categories in two- to five-year-olds [12,45,48,50,52,53]. These were based on four distinct samples, which included three community samples [12,48,50] and one clinical sample [52,53]. One study was judged to have a higher risk of bias [45] and five were judged to have a lower risk of bias [12,48,50,52,53].

One study used Moss’ French-Canadian sample and analyzed concurrent associations between maternal depressive symptoms and attachment among children aged three to four years [48]. Results indicated that mothers of avoidant, secure, and ambivalent children reported similar levels of depression symptoms. As no significant differences were found between the attachment groups, we conducted post-hoc calculations of between-group effect sizes to examine the direction and magnitude of differences between classifications. This revealed that mothers of disorganized/controlling children tended to report higher depression scores than mothers of secure children, with an overall effect in the small range (*d* = 0.35). Two studies used the 36-month wave of the NICHD SECCYD sample [12,50]. Within this sample, mothers of disorganized/controlling children reported the highest levels of depression, with significant differences found between these mothers and mothers of secure children (*d* = 0.22) [50]. Differences between the secure group and the avoidant/ambivalent groups occurred on a smaller scale, with effect sizes ranging from 0.07 (avoidant vs. secure) to 0.11 (ambivalent vs. secure). When the disorganized/controlling group was further sub-divided into behaviourally-disorganized, controlling-caregiving, controlling-punitive, and controlling-mixed [12], the behaviourally-disorganized sub-group was the only sub-group that significantly differed from the secure group in terms of maternal depression scores (*d* = 0.40). A study of 4.5-year-old children and their mothers found that mothers of ambivalent children reported the most depressive symptoms, relative to mothers of secure, avoidant, and controlling children [54]. Meanwhile, differences among the avoidant, secure, and disorganized/controlling groups were non-significant. It should be noted that, in this study, behaviourally-disorganized children were forced into the avoidant, secure, or ambivalent categories rather than grouped with the controlling category as is typically done.

Two studies investigated the same sample of mothers diagnosed with depression compared to a non-depressed control group and compared the distribution of attachment classifications across groups [52,53]. Results indicated that there was a significantly lower proportion of secure children and a significantly higher proportion of disorganized/controlling children with depressed mothers (vs. non-depressed mothers). The proportions of avoidant and ambivalent children were similar across groups.

1.1. Summary: Maternal depressive symptoms and attachment in 2- to 5-year-olds (Concurrent associations). Nine studies were quantitatively [12,44–48,52] and/or qualitatively [12,45,48,50,52,53] synthesized in this section. Summarizing results across attachment categorizations and syntheses, it was found that significant differences exist among attachment categories as a function of concurrently measured maternal depressive symptoms. Relative to the other attachment classifications, and to organized attachments generally, disorganized/controlling attachment is most consistently associated with higher levels of maternal depressive symptoms. Although one study did not find this trend, this study forced behaviourally-disorganized children into their best-fitting organized (i.e., secure, avoidant, or ambivalent) category. Given that mothers of behaviourally-disorganized children in the NICHD SECCYD sample were found to report the most depressive symptoms relative to all other categories and sub-categories of D, this incongruent result may be attributable to methodological differences.

Associations between attachment insecurity and maternal depressive symptoms were not robust. Although results of the meta-analysis were significant, results of diagnostic analyses suggest that a significant degree of publication bias may be present.

#### 1.2. Maternal depressive symptoms and attachment in 2- to 5-year-olds: Longitudinal associations

1.2.1. Secure vs. Insecure: Qualitative synthesis. Only two studies reported on longitudinal associations between maternal depressive symptoms and attachment insecurity in two- to five-year-olds [42,51]. As a result, these studies were synthesized qualitatively. These were based on one clinical sample [42] and one community sample [51]. Both samples were judged to have a lower risk of bias. In a study that oversampled children with prenatal drug exposure [42], maternal depressive symptoms measured at child age four months did not differ among dyads categorized as secure and insecure at 36 months. The other study used the NICHD SECCYD sample and applied clinical cut-offs to mothers’ self-reported depression symptom scores (assessed at 1, 6, 15, 24, and 36 months) to delineate trajectories of maternal depressive symptoms over the child’s first three years of life [51]. Findings indicated that mothers with intermittent (i.e., elevated symptoms at least twice, separated by a period of lower scores) and chronic (i.e., elevated symptoms at least three out of five times) were more likely to have children categorized as insecure, compared to mothers who had not reported elevated depressive symptoms since the birth of their child (intermittent vs. never, *d* = 0.38; chronic vs. never, *d* = 0.24).

1.2.2. Organized vs. Disorganized/controlling: Qualitative synthesis. Three studies reported on longitudinal associations between maternal depressive symptoms and attachment disorganization in two- to five-year-olds [42,43,47]. These were based on two distinct samples, and therefore results were synthesized qualitatively. All three studies were judged to have a lower risk of bias. In a study that oversampled children with prenatal drug exposure [42], maternal depressive symptoms measured at child age four months did not differ among dyads categorized as organized and disorganized/controlling at 36 months. Within the other sample, null effects were found when comparing disorganized/controlling and organized 3-year-old children based on maternal depression assessed prenatally [43,47], and at 6, 12, and 24 months [43], after controlling for maternal education and age at child’s birth. This study was based on a sample in which low birthweight infants and mothers undergoing treatment for anxiety or depression were over-represented.

1.2.3. A/B/C/D Categories: Qualitative synthesis. Four studies reported on longitudinal associations between maternal depressive symptoms and attachment categories in two- to five-year-olds [42,50,51,55]. These were based on two unique samples, including one community sample and one clinical sample. One study was judged to have a higher risk of bias [55] and the others were judged to have a lower risk of bias. Because the higher risk of bias study [55] used a smaller, randomly selected subsample of the NICHD SECCYD sample and there were already two studies based on the full sample in this outcome category [50,51], it was not described below.

Two studies examined longitudinal associations between maternal depressive symptoms and attachment at age three in the full NICHD SECCYD sample [50,51]. The first study, based on the full sample of 1,140, found that mothers of disorganized/controlling children reported significantly more depressive symptoms than mothers of secure children, whereas mothers of avoidant and ambivalent children reported similar symptom levels [50]. When the earlier waves of this study (i.e., 6 months, 15 months, 24 months) were each analyzed separately, mothers of children classified as disorganized/controlling at 36 months consistently reported the most depressive symptoms, whereas mothers of avoidant, ambivalent, and secure children reported similar symptom levels across all time points. Comparing mothers of disorganized/controlling children to mothers of secure children, all effect sizes were in the small range (*d* = 0.21–0.33), with the largest differences seen at the 24-month time point. Another NICHD SECCYD study applied clinical cut-offs to mothers’ self-reported depressive symptom scores to delineate trajectories of maternal depressive symptoms over the child’s first three years of life [51]. Mothers whose self-reports met clinical cut-offs at least once since the birth of their child (i.e., at 1, 6, 15, 24, or 36 months) were more likely to have a child categorized as disorganized/controlling at age three. In a follow-up analysis in which demographic variables (i.e., maternal education, partner status, and child gender) were controlled for, additional findings emerged. Mothers with intermittent (i.e., elevated symptoms at least twice, separated by a period of lower scores) depressive symptoms were more likely to have ambivalent or disorganized/controlling children, compared to mothers who never reported elevated symptoms. In addition, mothers who reported chronic (i.e., elevated symptoms at least three out of five times) depressive symptoms were more likely to have disorganized/controlling children, compared to mothers who never reported elevated symptoms. Finally, mothers who reported elevated symptoms early in their child’s life (i.e., elevated symptoms at 1-, 6-, and/or 15-months, but not after) were less likely to have avoidant children compared to mothers who never reported elevated symptoms. One study oversampled children with prenatal drug exposure [42], and found that maternal depressive symptoms measured at child age four months did not significantly differ among mothers of secure, avoidant, ambivalent, and disorganized/controlling children.

1.2. Summary: Maternal depressive symptoms and attachment in 2- to 5-year-olds (Longitudinal associations). Five studies were qualitatively synthesized in this section [42,43,47,50,51]. Summarizing results across attachment categorizations and syntheses revealed mixed findings. Results from the NICHD SECCYD suggest that mothers of disorganized/controlling children consistently report the most depressive symptoms across the child’s first three years. Dichotomizing maternal depression self-reports based on clinical cut-offs revealed that the trajectory of elevated maternal depressive symptoms throughout the child’s life had unique associations with attachment outcomes; mothers who had persistent elevations were more likely to have children who were classified as insecurely attached generally, and with disorganized/controlling or ambivalent attachment specifically. These trends were significant even after controlling for maternal education and partner status and child gender. Conversely, two large clinical/risk samples did not find differences in maternal depressive symptoms across attachment groups [42] or as a function of attachment insecurity or disorganization [42,43,47]. However, these both examined associations with maternal depression scores at specific time points, rather than examining patterns across time. Based on these findings, it can be concluded that persistent trajectories of maternal depressive symptoms in normative populations are likely to have implications for children’s attachment behaviours at early preschool-age. However, it is unclear whether the same pattern holds for at-risk and clinical samples.

### 2. Maternal depressive symptoms and attachment in 5- to 7-year-olds

Only four studies examined associations between maternal depressive symptoms and attachment in five- to seven-year-old children [56–59] using the coding system by Main and Cassidy [9].

#### 2.1. Maternal depressive symptoms and attachment in 5- to 7-year-olds: Concurrent associations

2.1.1. A/B/C/D categories: Qualitative synthesis. Two studies from the same sample examined concurrent relationships between maternal depressive symptoms and attachment categories in five- to seven-year-old children [57,58]. One was rated as having a higher risk of bias [58].

The first study [58], based on a sample of 121 mother-child dyads, found that self-reported maternal depressive symptoms varied significantly among categories. As between-group contrasts were not conducted by the authors, we conducted a post-hoc calculation of between-group effect sizes to examine the direction and magnitude of differences between classifications. This showed that mothers of disorganized/controlling children reported the *fewest* depressive symptoms, (*d* = 0.16, compared to secure group) and mothers of ambivalent children reported the most depressive symptoms (*d* = 0.6, compared to secure group). When the disorganized/controlling group was further sub-divided (i.e., controlling-caregiving, controlling-punitive, behaviorally disorganized) in a larger sample from the same longitudinal study [48], no significant differences were identified across the six classifications (i.e., A/B/C/Controlling-caregiving/Controlling-punitive/Behaviourally-disorganized). When a post-hoc calculation of between-group effect sizes was conducted, mothers of controlling-caregiving children were found to report the least depressive symptoms, with an effect size in the small-moderate range, compared to the secure group (*d* = 0.5). Meanwhile, mothers of behaviourally-disorganized children reported the most depressive symptoms, with mean differences in the small range (*d* = 0.3), compared to the secure group. With this said, it should be noted that this study did not find statistically significant differences across groups. This is likely because each of the disorganized/controlling groups had 13 or fewer members, compared to the 139 children in the secure group.

2.1.2. Secure vs. Insecure-organized (A/C) vs. Disorganized/Controlling. Only one study combined the avoidant and ambivalent groups in order to perform a contrast of secure, insecure-organized, and disorganized/controlling groups [59], thus precluding quantitative synthesis. This study was judged to have a higher risk of bias. Results indicated no significant differences among these three groups based on maternal depressive symptoms measured at the same time point.

2.1. Summary: Maternal depressive symptoms and attachment in 5- to 7-year-olds (Concurrent associations). Three studies were qualitatively synthesized in this section [57–59]. Summarizing results across attachment categorizations and syntheses, no significant differences were found between attachment classifications of five- to seven-year-old children as a function of concurrent maternal depressive symptoms. An examination of trends in between-group differences (based on post-hoc effect size calculations) suggests that, within this single sample, mothers of behaviourally-disorganized children in this age group tended to report the most depressive symptoms.

#### 2.2. Maternal depressive symptoms and attachment in 5- to 7-year-olds: Longitudinal associations

2.2.1. A/B/C/D Categories: Qualitative synthesis. Two studies investigated longitudinal relationships between maternal depressive symptoms and attachment in five- to seven-year-old children [56,57], with one of these being judged as having a higher risk of bias [56]. One study investigated group differences among attachment classifications based on maternal depressive symptoms measured two years prior (i.e., at 3 to 5 years)[57]. Findings indicated no overall group differences between mothers of secure, avoidant, ambivalent, controlling-caregiving, controlling-punitive, and insecure-other children. Mothers of secure children reported the fewest depressive symptoms, while mothers of behaviourally-disorganized children reported the most, with effect sizes indicating a small size effect distinguishing these two groups (*d* = 0.37). The second study, based on the same sample, generated a dichotomous outcome for maternal depression (i.e., depressed/non-depressed) based on clinical cut-offs for the self-report measure [56]. Results indicated that the proportion of mothers who had experienced elevated depressive symptoms during the prior wave of the study (i.e., two years prior) did not differ among the four attachment groups during the follow-up wave.

2.2. Summary: Maternal depressive symptoms and attachment in five- to seven-year-olds (Longitudinal associations). Two studies were qualitatively synthesized in this section [56,57]. The results of these two studies, which used the same community sample, indicated no significant longitudinal associations between maternal depressive symptoms and attachment categories at age 5- to 7- years, regardless of whether maternal depression was operationalized as a continuous or dichotomous outcome (above/below clinical cut-off). However, trends indicated that depression levels tended to be higher among mothers whose children were later classified as behaviourally-disorganized, compared to mothers of children who were later classified as secure, with results indicating a small effect size.

## Discussion

To our knowledge, this is the first study to systematically review and meta-analyze the literature examining maternal depression symptoms as predictors and correlates of children’s attachment behaviour as assessed by the Cassidy and Marvin [10] and Main and Cassidy [9] attachment classification systems. Our findings demonstrate that higher levels of maternal depressive symptoms are consistently associated with disorganized/controlling child attachment throughout the preschool period. Relationships with insecure attachment in general were detected but cannot be deemed conclusive, due to study heterogeneity and publication bias. Our interpretation of these findings, along with implications for research and clinical practice, are discussed below.

### Associations between maternal depressive symptoms and attachment in 2- to 5-year-old children

Results of the meta-analysis examining associations between attachment insecurity and concurrent maternal depression levels were inconclusive. Despite a significant overall mean effect, diagnostic tests revealed a significant risk of publication bias, indicating that effects of the included published studies may not be representative of the true relationship between the two variables. However, the overall direction of the effect is consistent with results from Atkinson and colleagues’ meta-analysis [13], and indicated that maternal depression levels were generally higher among mothers of insecure children. It should be noted that this previous meta-analysis did not specifically examine publication bias, thus, it is possible that this bias had an equal (but unknown) impact on the previous study. This result may be better understood in the context of other findings from this section. Given that mothers of disorganized/controlling children reported the most depression symptoms regardless of whether a dichotomy or four-way classification was used, while mothers of secure, avoidant, and ambivalent children reported similar symptoms levels, it is logical to suggest that elevated scores in the disorganized/controlling group may be driving the group differences in both meta-analyses (Insecure vs. Secure and Disorganized/controlling vs. Organized).

With respect to the association between attachment disorganization and concurrent maternal depressive symptoms, results identified a significant and robust small effect, with mothers of disorganized/controlling children reporting higher depression levels than mothers of organized (i.e., avoidant/ambivalent/secure) children. This effect was not moderated by study quality or sample type, although effect sizes tended to be larger among clinical samples compared to normative samples. The size of this effect (*d* = 0.27) is larger than the effect identified in a previous meta-analysis on disorganized attachment (re-calculated as *d* = 0.12) [30]. However, the previous meta-analysis did not differentiate between studies that examined concurrent vs. longitudinal associations between maternal depression and attachment disorganization and collapsed across child age (with samples ranging in child age from 12 months to 54 months), two potential sources of heterogeneity that may have moderated the effect size. Given that use of the preschool attachment coding systems has increased considerably since the publication of the original meta-analysis, the more recent studies included in the present review are likely more in line with the current understanding of disorganized and controlling behaviours observed during the preschool period. While we did not find a significant moderating effect of sample type, effect sizes from the two clinical samples were relatively larger than effect sizes from community samples and were in the expected direction (i.e., higher depression levels in disorganized/controlling group), a trend which is consistent with previous meta-analyses [30,60]. Collectively, these results provide strong evidence suggesting that disorganized/controlling attachment has the strongest and most consistent associations with concurrent maternal depressive symptomology, with effect sizes across studies consistently emerging in the small range.

Generally, longitudinal associations between maternal depressive symptoms and preschooler attachment were less frequently examined but tended to be less robust in cases where they were examined. Studies that used clinical samples and had longer gaps between the assessment of maternal depression and preschooler attachment tended to have particularly small effect sizes. A key finding to emerge from the longitudinal syntheses was the added value of examining trajectories of maternal depressive symptoms, rather than averaging scores across time. While self-reported depressive symptoms have been described as a distinct clinical phenomenon from clinical depression [61], this finding provides some indication that documenting patterns in symptoms over time (rather than at a single time point) may be a more clinically-relevant use of this type of measure.

### Associations between maternal depressive symptoms and attachment in 5- to 7-year-old children

All findings related to attachment among five- to seven-year-old children were based on Moss’ French-Canadian sample. Across both concurrent and longitudinal analyses, significant relationships between maternal depressive symptoms and attachment outcomes were not identified. However, the direction and magnitude of effect sizes were consistent with findings from the earlier age group and indicated that mothers of behaviourally-disorganized children tended to report the highest levels of depressive symptoms. This suggests a degree of developmental continuity in terms of between-group differences and gives added empirical support to the notion that maternal depressive symptoms are most strongly linked to disorganized/controlling attachment.

Collectively, our syntheses showed that maternal depressive symptoms were more consistently associated with disorganized/controlling (rather than insecure) attachment behaviours both concurrently and longitudinally. From a theoretical perspective, the presence of frightening or disrupted parent behaviours differentiates disorganized/controlling mother-child attachment relationships from other forms of insecure attachment [62]. Avoidant and ambivalent attachment behaviours are thought to occur when the child does not trust the parent’s capacity to support their needs in a distressing context and thus adapts to this non-optimal parenting by exaggerating (ambivalent) or minimizing (avoidant) their expression of distress [9,10]. Conversely, in disorganized/controlling attachment relationships, the child is hypothesized to fear the parent’s reaction to the child’s distress and either adapts (i.e., controlling behaviours) or engages in anomalous (i.e., behaviourally-disorganized) behaviours [9]. Our results suggest that maternal depressive symptoms may impact the attachment relationship not only by reducing the mother’s availability to the child, but also by inducing fear. More research will be needed to understand the specific maternal behaviours that occur in the context of the attachment relationship as a consequence of maternal depression and depressive symptoms.

### Limitations

Our results should be viewed in the context of some potential limitations. First, despite our comprehensive and systematic search strategy, it is possible that some relevant articles were omitted from this review. However, we are confident that our strategy of screening the more ambiguous abstracts and selectively including articles among them that were authored by key attachment researchers ensured that the key articles on this topic were captured in our final set of included studies. Second, most of the effect sizes descriptively reported in this review do not control for variables known to be associated with attachment categorization (e.g., child gender, family socioeconomic status), which is a limitation of conducting secondary data-analysis. However, the fact that significant effects were still found in some instances where these variables were controlled for (e.g., [51]) gives an indication that a true effect may exist independently of these moderating variables. Third, there was a limited degree of sample heterogeneity among the included studies, with the majority of studies being based on middle- to high-socioeconomic status, primarily Caucasian, two-parent North American families. This may limit the generalizability of our findings to more socioeconomically diverse samples with alternative family compositions. Finally, while our findings support an association between maternal depressive symptoms and disorganized/controlling child attachment behaviours, no conclusions can be drawn concerning the directionality of this effect. While it is possible that higher levels of maternal depressive symptoms contribute to parenting behaviours that increase the likelihood of disorganized and controlling child attachment behaviours, it is equally possible that having a child who engages in disorganized/controlling attachment behaviours contributes to or exacerbates depressive symptoms in the mother.

### Implications for research and clinical practice

The results of this study have important implications for clinical practice and future research. A core tenet of attachment theory is that caregiver behaviour during caregiver-child interactions is a primary determinant of children’s attachment patterns. By identifying a significant relationship between maternal depressive symptoms (in both clinical and non-clinical samples) and preschool attachment disorganization, we have made progress towards understanding the ways in which maternal psychological challenges may be associated with maladaptive attachment outcomes. Future research can build on these findings by investigating behavioural manifestations of depression that may occur in the context of the attachment relationship, and by examining the impact of depression and other mental health challenges in non-maternal caregivers (e.g., fathers).

Our findings suggest that it is important for future research investigating maternal mental health as a correlate of attachment to avoid over-simplifying attachment outcomes by focusing on a secure-insecure dichotomy. Given that the most pronounced differences among attachment groups were detected when the disorganized/controlling group was examined separately from the other categories, we can reasonably assume that collapsing across the insecure groups may result in the masking of important group differences. An interesting area of future exploration will be to work towards a better understanding of the within-disorganized/controlling group variation. Since studies from two large samples found that mothers of behaviourally-disorganized children consistently reported the most depressive symptoms, it is possible that distinct patterns of maternal behaviour may place children at elevated risk for this particular attachment outcome.

Disorganized attachment has been associated with a host of unfavourable outcomes in the realm of children’s social and emotional development [4,30,63]. Thus, a better understanding of the correlates and predictors of these attachment behaviours is a step towards being able to identify and prevent the maladaptive developmental outcomes associated with this pattern of mother-child interactions. One of the findings discussed in this review, which emerged from the NICHD SECCYD sample, was that chronic and intermittent depressive symptoms throughout the child’s first three years of life were associated with an increased risk of disorganized/controlling attachment at age three. While other significant predictors of disorganized/controlling attachment have been identified in the literature (e.g., maltreatment [30], frightening or anomalous behaviour [64]), many are subtle caregiver behaviours that are not readily observable. In contrast, there are opportunities to screen for maternal mental health challenges during the early years of a child’s life, such as during postnatal or well-baby visits, which have been implemented with success in some regions of Canada and the United States [65]. The findings of this review lend further support to the utility of maternal mental health screening during the early years as a strategy for optimizing child development outcomes.

In conclusion, our findings represent an important step towards a better understanding of the correlates and predictors of disorganized/controlling attachment and highlight the need for future research of more rigorous methodological quality to further elucidate how maternal depression and other mental health challenges may contribute directly and indirectly to attachment in young children.

## Supporting information

S1 Appendix PRISMA checklist. (PDF)

S2 Appendix PsycINFO search strategy. (PDF)

S3 Appendix Protocol for ambiguous abstracts. (PDF)

S4 Appendix Risk of bias tool. (PDF)

S5 Appendix Meta-analysis dataset. (XLSX)
