# Supplementary material for: Associations between mother-preschooler attachment and maternal depression symptoms: A systematic review and meta-analysis
Source: PLoS One. 2018 Oct 2;13(10):e0204374. doi: 10.1371/journal.pone.0204374 (PMC6168129; doi:10.1371/journal.pone.0204374)
Supplement: S2 Appendix — (PDF) [file pone.0204374.s002.pdf]

Supplementary Appendix 2. PsycINFO Search Strategy.

Database: PsycINFO
Search Strategy:
----
1 attachment behavior/
2 attachment theory/
3 attachment*.tw.
4 1 or 2 or 3
5 (separation* adj7 reunion*).mp.
6 strange situation*.mp.
7 Preschool Attachment Classification System*.mp.
8 PACS.mp.
9 cassidy.af.
10 MacArthur.af.
11 Attachment Working Group.af.
12 Organized.mp.
13 Disorganized.mp.
14 Disorganization.mp.
15 Controlling.mp.
16 Insecur*.mp.
17 Secure.mp.
18 Security*.mp.
19 Avoidant*.mp.
20 Avoidance.mp.
21 Ambivalent.mp.
22 Dependent.mp.
23 Resistant.mp.
24 5 or 6 or 7 or 8 or 9 or 10 or 11 or 12 or 13 or 14 or 15 or 16 or 17 or 18 or 19 or 20 or 21 or 22 or 23
25 (infan* or baby* or babies or child* or toddler* or schoolchild* or school child* or school age* or pre-school or preschool* or nursery school* or kindergar* or primary school* or elementary school*).mp.
26 4 and 24 and 25
27 limit 26 to all journals
