# Supplementary material for: Associations between mother-preschooler attachment and maternal depression symptoms: A systematic review and meta-analysis
Source: PLoS One. 2018 Oct 2;13(10):e0204374. doi: 10.1371/journal.pone.0204374 (PMC6168129; doi:10.1371/journal.pone.0204374)
Supplement: S3 Appendix — (PDF) [file pone.0204374.s003.pdf]

Supplementary Appendix 3. Protocol for ambiguous abstracts.

Following abstract review, there was a significant proportion of studies for which key methodological details were unclear, based on the abstract (*e.g.,* age of attachment measure/coding system not clearly specified). Thus, the following criteria were used to identify relevant studies among these studies. If either one of these criteria was met, the study was retained for full-text review.

1. Authored by individuals identified to contribute to the development of the preschool attachment coding manuals (Cassidy, Marvin, & MacArthur Attachment Working Group, 1992; Main & Cassidy, 1988).
2. Authored by key authors in the field of child attachment (see accompanying table below)
3. Completed using the National Institute of Child Health and Development (NICHD) data

The following is a table of authors identified as key authors in the field of attachment, with accompanying rationale:

**Author**	**Justification**
Cassidy	Authored kindergarten attachment coding guidelines; Authored Preschool Attachment Classification System guidelines
Main	Authored kindergarten attachment coding guidelines; Contributor to Preschool Attachment Classification System guidelines
Marvin	Authored the Preschool Attachment Classification System guidelines
MacArthur Attachment Working Group	Authored the Preschool Attachment Classification System guidelines
Ainsworth	Contributor to Preschool Attachment Classification System guidelines
Beckwith	Contributor to Preschool Attachment Classification System guidelines
Belsky	Contributor to Preschool Attachment Classification System guidelines
Booth	Contributor to Preschool Attachment Classification System guidelines
Bronson	Contributor to Preschool Attachment Classification System guidelines
Crnic	Contributor to Preschool Attachment Classification System guidelines
Easterbrooks	Contributor to Preschool Attachment Classification System guidelines
Greenberg	Contributor to Preschool Attachment Classification System guidelines
LaGasse	Contributor to Preschool Attachment Classification System guidelines
Ridgeway	Contributor to Preschool Attachment Classification System guidelines
Barnard	Contributor to Preschool Attachment Classification System guidelines
Beeghly	Contributor to Preschool Attachment Classification System guidelines
Blacher	Contributor to Preschool Attachment Classification System guidelines
Bretherton	Contributor to Preschool Attachment Classification System guidelines
Carmichael-Olsen	Contributor to Preschool Attachment Classification System guidelines
Cicchetti	Contributor to Preschool Attachment Classification System guidelines
Cummings	Contributor to Preschool Attachment Classification System guidelines
Gottman	Contributor to Preschool Attachment Classification System guidelines

Harmon	Contributor to Preschool Attachment Classification System guidelines
Morisset	Contributor to Preschool Attachment Classification System guidelines
Slough	Contributor to Preschool Attachment Classification System guidelines
Spieker	Contributor to Preschool Attachment Classification System guidelines
Stevenson-Hinde	Contributor to Preschool Attachment Classification System guidelines
Speltz	Contributor to Preschool Attachment Classification System guidelines
Purcell	Contributor to Preschool Attachment Classification System guidelines
Bailey	Key researcher in field of attachment
Bernier	Key researcher in field of attachment
Bureau	Key researcher in field of attachment
Crittenden	Key researcher in field of attachment
Cyr	Key researcher in field of attachment
Dubois	Key researcher in field of attachment
Howes	Key researcher in field of attachment
Humber	Key researcher in field of attachment
Lecompte	Key researcher in field of attachment
Lyons-Ruth	Key researcher in field of attachment
McCartney	Key researcher in field of attachment
McElwain	Key researcher in field of attachment
Mongeau	Key researcher in field of attachment
Moran	Key researcher in field of attachment
Moss	Key researcher in field of attachment
O'Connor	Key researcher in field of attachment
Parent	Key researcher in field of attachment
Pascuzzo	Key researcher in field of attachment
Pederson	Key researcher in field of attachment
Pierrehumbert	Key researcher in field of attachment
Rousseau	Key researcher in field of attachment
Solomon	Key researcher in field of attachment
Sroufe	Key researcher in field of attachment
St-Laurent	Key researcher in field of attachment
Tarabulsy	Key researcher in field of attachment
Van Ijzendoorn	Key researcher in field of attachment
NICHD, National Institute of Child Health and Development	Key collaboration in field of attachment
