# Supplementary material for: Associations between mother-preschooler attachment and maternal depression symptoms: A systematic review and meta-analysis
Source: PLoS One. 2018 Oct 2;13(10):e0204374. doi: 10.1371/journal.pone.0204374 (PMC6168129; doi:10.1371/journal.pone.0204374)
Supplement: S4 Appendix — (PDF) [file pone.0204374.s004.pdf]

Supplementary Appendix 4. Risk of Bias Tool.

1. Was the research question or objective in this paper clearly stated?
2. Was the study population clearly specified and defined?
3. Was the participation rate of eligible persons at least 50%?
4.
   a) Were all the subjects selected or recruited from the same or similar populations (including the same time period)?
   b) Were inclusion and exclusion criteria for being in the study pre-specified and applied uniformly to all participants?
5. Was a sample size justification, power description, variance accounted for or effect estimates provided?*
6. For predictor variables (i.e., maternal depression) that can vary in amount or level, did the study examine different levels of the predictor as related to the outcome (i.e., PACS) (e.g., categories of depression levels, or depression measured as continuous variable)?
7. Were the predictor variables (i.e., maternal depression) clearly defined, valid, reliable, and implemented consistently across all study participants?*
8. Was the predictor variable (i.e., maternal depression) assessed more than once over time?
9. Were measures of the outcome variable (i.e., PACS) clearly defined, valid, reliable, and implemented consistently across all study participants?*
10. Were the outcome assessors (i.e., PACS coders) blinded to the exposure (clinical) status of participants?*
11. Was loss to follow-up after baseline 20% or less (i.e., was the retention rate > 80%)?*
12. Were key potential confounding variables measured and adjusted statistically for their impact on the relationship between exposure(s) and outcome(s)?*
13. Is the distribution of the overall study population by gender described?
14. Are the statistical methods described?
15. Have actual probability values been reported (e.g., 0.035 rather than < 0.05) for the main outcomes except where the probability value is less than 0.001?

*indicates item prioritized for risk of bias judgment.
